# Green Synthesis, Characterization and Bioactivity of *Mangifera indica* Seed-Wrapped Zinc Oxide Nanoparticles

**DOI:** 10.3390/molecules28062818

**Published:** 2023-03-21

**Authors:** Shanmugam Rajeshkumar, Royapuram Parthasarathy Parameswari, Dayalan Sandhiya, Khalid A. Al-Ghanim, Marcello Nicoletti, Marimuthu Govindarajan

**Affiliations:** 1 Department of Pharmacology, Saveetha Dental College and Hospitals, Saveetha University, SIMATS, Chennai 600077, TN, India; 2 Department of Medicinal and Applied Chemistry, Kaohsiung Medical University, Kaohsiung City 80708, Taiwan; 3 Department of Zoology, College of Science, King Saud University, Riyadh 11451, Saudi Arabia; 4 Department of Environmental Biology, Sapienza University of Rome, 00185 Rome, Italy; 5 Unit of Vector Control, Phytochemistry and Nanotechnology, Department of Zoology, Annamalai University, Annamalainagar 608002, TN, India; 6 Department of Zoology, Government College for Women (Autonomous), Kumbakonam 612001, TN, India

**Keywords:** nanotechnology, ZnO NPs, biofabrication, antioxidant, antibacterial activity

## Abstract

In the realm of nanoparticles, metal-based nanoparticles have traditionally been regarded as the pioneering category. Compared to other nanoparticles, zinc oxide nanoparticles have several advantages, including optical and biological properties, which provide them a significant competitive advantage in clinical and biological applications. In the current investigation, we used an aqueous *Mangifera indica* seed extract to synthesize nanoparticles of zinc oxide (ZnO NPs). UV-Vis spectroscopy, Fourier transform infrared spectroscopy analysis, atomic force spectroscopy, X-ray diffraction, scanning electron microscopy, and transmission electron microscopy were used to characterize the synthesized ZnO NPs. The nanoparticles were assessed for their potential to inhibit bacterial growth and protect cells from free radical damage. According to the current study’s findings, zinc oxide nanoparticles that had been modified with the aid of mango seeds were very efficient in preventing the development of the tested bacteria and were also powerful antioxidants.

## 1. Introduction

Nanotechnology has been gaining attention recently as a potential platform for future growth in several fields. Nanotechnology has drawn significant attention in the healthcare, engineering and food industries by offering novel prospects in the respective fields. In particular, theranostics, a cutting-edge combination system of therapeutics and diagnostics, utilize nanotechnology principles for target-specific drug delivery and enhanced bioavailability of active pharmaceutical ingredients [1,2]. The field of nanotechnology deals with various synthesis methods, particle size reformations and structural variations of nanoparticles. Nanoparticles are nanosized materials ranging in size <100 nm with high thermal stability, high surface-to-volume ratio, high electrical, mechanical, optical as well as magnetic properties [3].

In the last decade, the use of nanoparticles has been the most significant archetype advancement in engineering, medicine and technology [4]. Nanoparticles may be classified as organic and inorganic nanoparticles. While metals and metal-derived oxide nanoparticles come under the inorganic nanoparticles classification, organic nanoparticles include solid lipid nanoparticles, polymeric nanoparticles, lipid-based nanocarriers, liposomes and carbon-based nanomaterials [5]. Metal nanoparticles are promise for site-specific drug administration, clinical diagnostics, bio-imaging, dental implants, and biomedicine due to their selectivity, sensitivity, optical, and electrical capabilities [6,7]. The method used to synthesize metal nanoparticles is an important key factor. Synthesis of metal nanoparticles may be accomplished using a wide variety of physical and chemical methods, including sol-gel, chemical reduction, hydrothermal, laser ablation, ion sputtering, etc., [8,9]. However, these methods encounter many downsides, including cost-expensive, instrumentations, skilled labour and environmental toxicity. Therefore, the green synthesis method has become an optimal method of choice for nanoparticles, wherein plants and microorganisms are used [9,10]. Furthermore, the green synthesis method of nanoparticle preparation has been considered eco-friendly and safer due to its stabilizing and reducing potentials [10]. Many different metals, including silver (Ag) [10], gold (Au) [11], copper (Cu) [12] and trace elements [13], have been used as reduction and coating agents for the fabrication of nanoparticles. Nevertheless, Ag, Au and Cu nanoparticles have been reported for their toxicity and consequent limit in clinical applications [10,11,12].

Zinc oxide (ZnO) is a rare, inorganic metallic oxide that has received significant attention as a safe, biocompatible and economical material. US FDA has approved ZnO as the safest metal oxide [14]. Zinc is best recognized for maintaining protein and nucleic acid interactions in cells and tissues. As compared to other physiologic metals like iron, cobalt, and manganese, ZnO’s chemical stability is far superior [15]. Zinc oxide nanoparticles (ZnO NPs) possess a wide range of engineering applications, such as catalysis, a piezoelectric device, pigments, chemical sensors, bio-molecular detection, diagnostic, cosmetic material and especially for UV protection [16,17,18,19,20,21,22]. Additionally, ZnO NPs have been demonstrated to possess anticancer, antimicrobial, anti-inflammatory and antidiabetic properties [23,24,25,26]. As ZnO is considered safe and exhibits significant antimicrobial properties, it has the potential against infectious diseases [26]. On exposure to metal nanoparticles, the bacterial cell membrane undergoes depolarization due to adsorption, resulting in permeability changes in the bacterial cell wall. Further, the changes lead to the free radical formation and thus cause membrane damage resulting in antibacterial or bactericidal activity [27,28].

Green nanotechnology has originated from green chemistry, which seeks to synthesize metal nanoparticles using medicinal plants. Medicinal plants are reliable sources of several chemical components needed to synthesize metallic nanoparticles [29], *viz*. polyphenols, flavonoids, alkaloids, terpenes, etc., which act as potent agents to reduce the metal from ionic state into its respective oxide forms during the process [8,30]. Phytochemicals found in medicinal plants have replaced the reducing agents’ sodium citrate, ascorbate and sodium borohydride in the chemical manufacturing technique of nanoparticles. As a result, the potentially harmful effects of chemical-reducing agents on the environment will be mitigated [29,31]. As a result, scientists are increasingly interested in developing methods for creating metal-based nanoparticles from plant extracts that are benign to the surrounding ecosystem.

The biosynthesis of ZnO NPs has been shown utilizing several plant extracts, including *Lobelia leschenaultiana* [32], *Agathos mabetulina* [33], *Laurus nobilis* [34], *Moringa olifera* [35], *Acalypha indica* [36], *Aspalathus linearis* [37], *Carica papaya* [38], green tea leaves [39], *Euphorbia jatropa* latex [40], *Andrographis paniculata* [41], *Chamaecostus cuspidatus* [42]. Compared to the above sources, the advantage of preparing ZnO NPs using mango seed extracts has been cost-effectiveness, as rinds and seeds are considered waste materials. However, the mango seed powder contains protein, oil, crude fiber and carbohydrate and is also enriched in potassium, magnesium, phosphorus, calcium and sodium. Non-essential amino acids such as arginine and glutamic acid and essential amino acids such as valine and phenylalanine are also present. In addition, it has other vitamins, including different Vitamin B (1, 2, 6 and 12), Vitamin C and Vitamin K. The nutritious value of mango seed powder is much higher [43]. Mangoes were used in the synthesis of zinc oxide nanoparticles, an economically and ecologically sound breakthrough [44,45]. Hence, our work synthesized ZnO NPs using mango seed extract. The structure and shape of synthesized ZnO NPs were investigated using a variety of techniques, ultraviolet-visible spectroscopy (UV-vis.), Fourier transform infrared (FT-IR), X-ray diffraction (XRD), scanning electron microscopy (SEM), transmission electron microscopy (TEM), and including atomic force microscopy (AFM). The antibacterial and antioxidant activities of ZnO NPs that were produced through green synthesis have also been investigated.

## 2. Results

### 2.1. UV-Visible Spectroscopy

**Figure 1:**
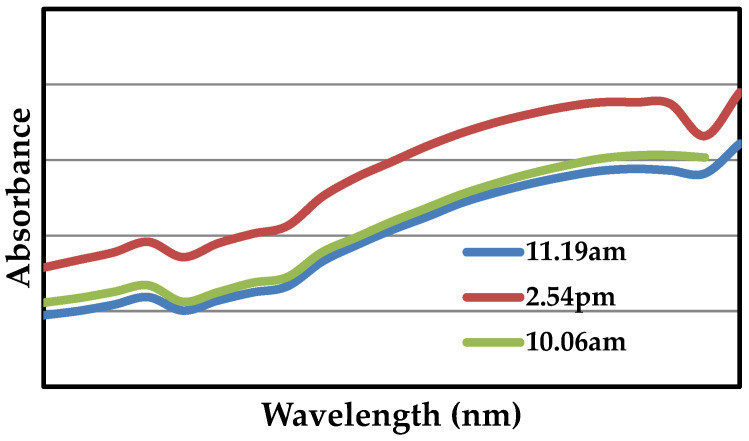
UV-Visible Spectrum of synthesized ZnO NPs using *Magnifera indica* seed extract.

As shown in Figure 1, ZnO NPs prepared with mango seeds powder were recorded using UV-Visible spectroscopy. UV-Visible spectra measurements were carried out at various time intervals of 4, 12 and 24 h. The absorbance of ZnO NPs rose gradually over 23 h, as evidenced by a linear relationship between the first value and subsequent readings performed at 1 h 13 min, 3 h 35 min and 19 h 12 min. The formation of ZnO NPs was confirmed by the observation of maximal absorbance at 480 nm, which is the typical wavelength for this formation. The observed UV spectrum around 450 nm indicates that in the reduction process, the semiconducting property of ZnO has not been lost [46].

### 2.2. FT-IR Spectroscopy

**Figure 2:**
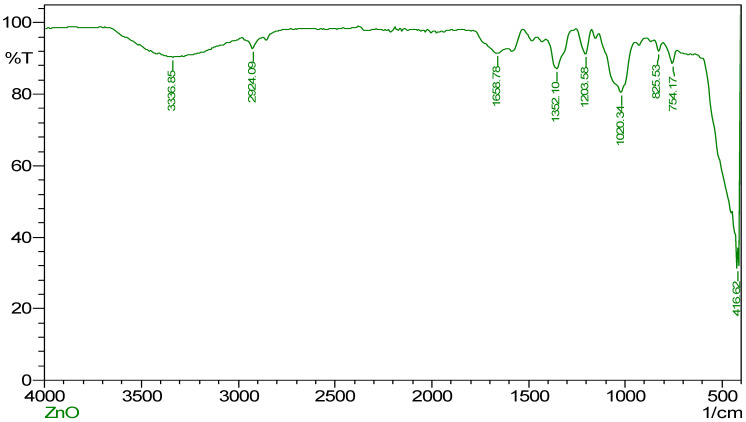
FT-IR spectrum of synthesized ZnO NPs using *M. indica* seed extract.

Characterization and an explanation of the functional groups involved in the synthesis, reduction, and stabilization of ZnO NPs were accomplished with the assistance of FT-IR spectroscopy. As shown in Figure 2, ZnO NPs may be mediated by extracts from mango seeds. Specifically, a prominent peak was detected at 3336.85 cm^−1^, analogous to the stretching vibration of −OH and –NH_2_ groups. These functional groups could be produced from the water and mango seed extract [8]. Further, another broad peak was observed at 2924.09 cm^−1^, corresponding to the carboxylic group’s O-H stretch. This peak can be seen in the spectrum. The peak at 1658.78 cm^−1^, which coincides with the C = O stretch that the ketone group has. The N = O bend of nitro groups is related to a peak located at 1352.10 cm^−1^, and peaks at 1203.58 cm^−1^ and 1020.34 cm^−1^, are associated with the C-O stretch of ester and ether groups, respectively. The peaks associated with aromatic groups are located at 825.53 cm^−1^, 754.17 cm^−1^, and 416.62 cm^−1^. Although numerous studies have reported using plant extracts for metal nanoparticle synthesis, the mechanism of bio-reduction is still elusive. On the other hand, the phytochemicals that are present in the plant source are the ones that are thought to be responsible for the nanoparticles’ reduction and stabilization [14,46]. In agreement, we speculate in the present study that the phytochemicals, such as polyphenols, flavonoids, and carotenes found in the seed extract, might be deeply involved in the bio-reduction of the nanoparticles [42,43].

### 2.3. Scanning and Transmission Electron Microscope (SEM)

**Figure 3:**
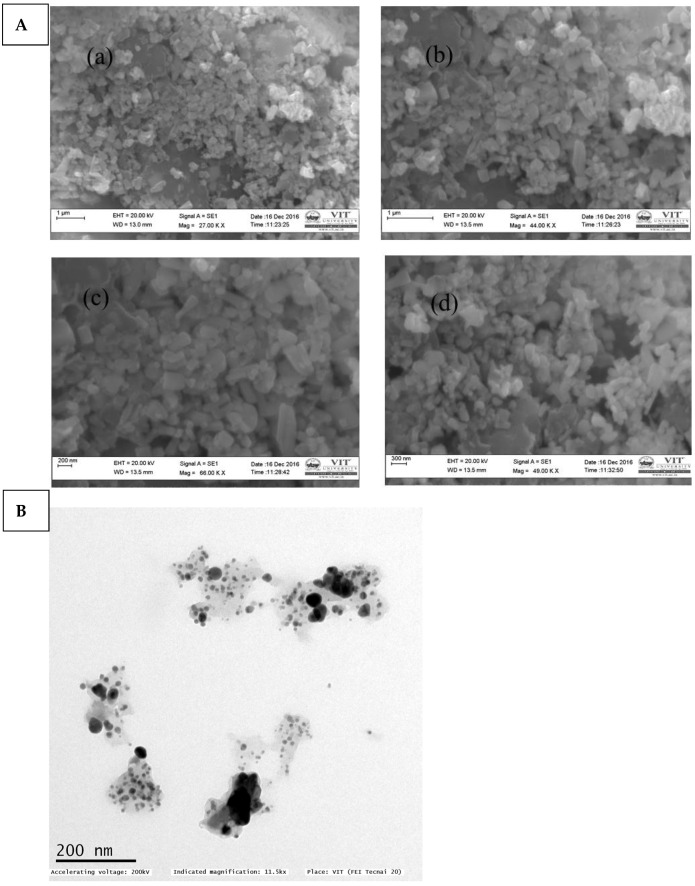
(**A**) SEM (Nanoparticle view under 27.00 KX (a), 44.00 KX (b), 66.00 KX (c), 49.00 KX (d) magnification) and (**B**) TEM study of ZnO NPs that were generated utilizing the extract of *M. indica* seeds.

The SEM was used in order to investigate the ZnO NPs’ morphology. Mango seeds mediated ZnO NPs surface morphology is seen in Figure 3. ZnO NPs powder was kept in a carbon-coated copper grid. The SEM image (Figure 3a) shows the presence of spherical, cylindrical, rectangle and triangle shape clustered nanoparticles with narrow size distribution. The images were captured at different magnifications of 27,000×, 44,000×, 49,000×, and 66,000×. Nanoparticles agglomerated, perhaps owing to mango seed extract phytoconstituents. The size of the nanoparticle was found to be approximately between 40 to 70 nm. Figure 3b shows TEM analysis of green synthesized ZnO NPs to confirm particle size. TEM confirmed that *M. indica* seed extract-mediated ZnO NPs were 40–60 nm.

### 2.4. EDAX Analysis

**Figure 4:**
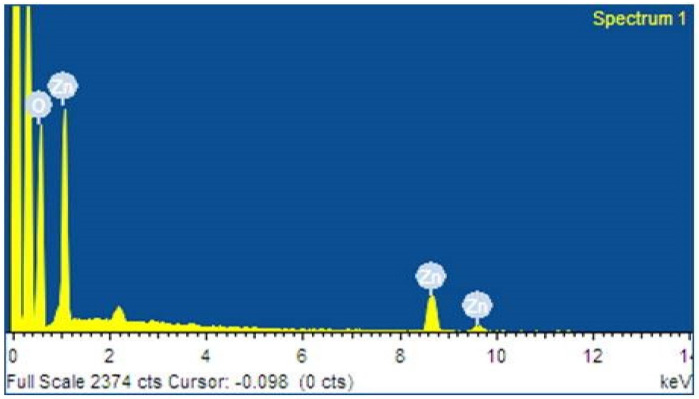
EDAX analysis of *M. indica* seed extract synthesized ZnO NPs.

Figure 4 shows the ZnO NPs’ EDAX elemental composition. Here, the seed-mediated ZnO NPs shows signals of Zinc, Oxygen and Carbon, as shown in the tabular column (Table 1). EDAX analysis also helps to identify the presence of any other compounds. It displays the energy in KeV. Furthermore, the EDAX spectra also help in confirming the pureness of the ZnO NPs synthesized from mango seed extract.

### 2.5. XRD Study of Generated ZnO NPs

**Figure 5:**
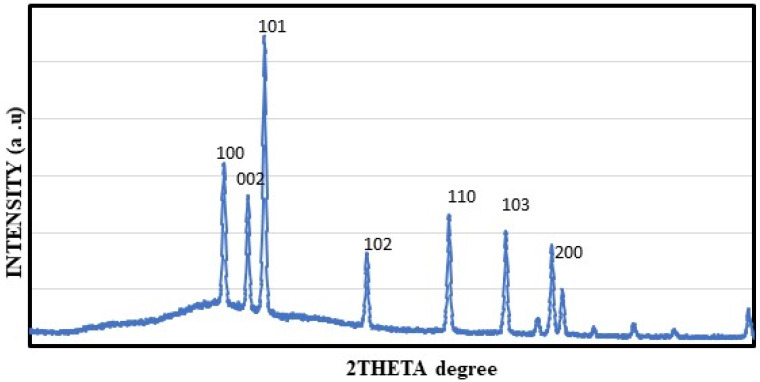
XRD analysis of *M. indica* seed extract synthesized ZnO NPs.

The XRD pattern of the synthesized ZnO NPs is shown in Figure 5. ZnO NPs were synthesized from mango seed extract, and their structure was characterized by XRD analysis. Analysis of ZnO NPs by XRD showed six different peaks between 10 and 90° in the 2θ value. The obtained ZnO NPs pattern was consistent with the XRD pattern published by the Joint Committee on Powdered Diffraction Standards (JCPDS file no: 85-1355). Meanwhile, peaks were shown with 2θ values of 31.4°, 34.0°, 35.9°, 47.1°, 56.2°, 62.5° and 68.7° which corresponds to (100), (002), (101), (102), (110), (103) and (200) respectively. ZnO NPs synthesized from *Andrographis paniculate, Chamaecostus cuspidatus* and *Agathosma betulina* showed an identical XRD pattern [33,41,42].

### 2.6. Atomic Force Microscopy (AFM)

**Figure 6:**
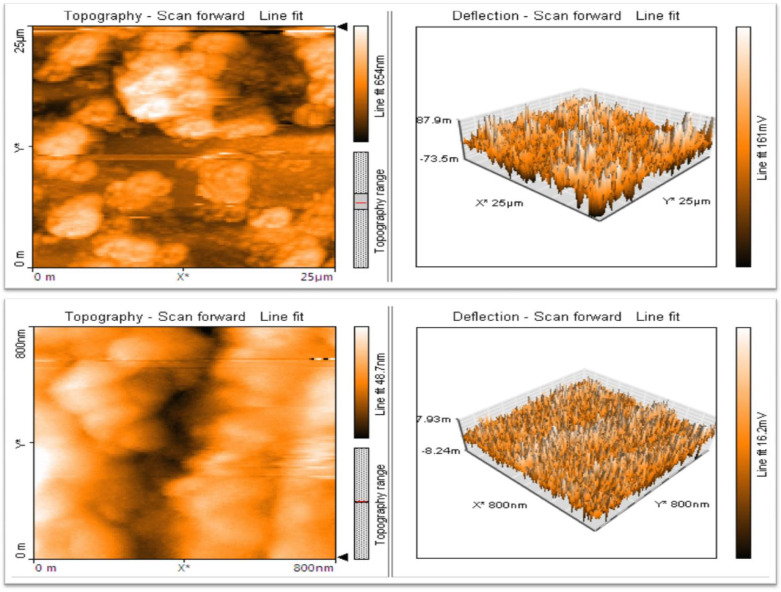
AFM images of *M. indica* seed extract synthesized ZnO NPs. (*—axis).

In Figure 6, the 3D images obtained by the AFM are reported. The outcome provided 2D and 3D imaging of biosynthesized ZnO NPs, revealing their average size to be 55 nm and their spherical form (Figure 6).

### 2.7. Antibacterial Activity

**Figure 7:**
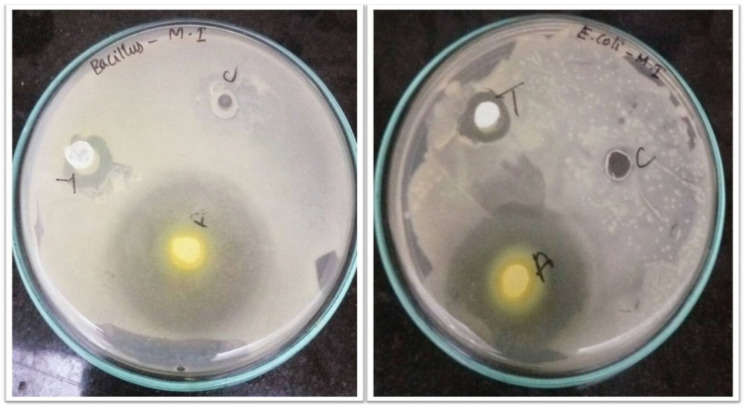
Synthesized ZnO NPs inhibit the growth of *B. subtilis* and *E. coli*. (A-*M. indica* seed-mediated ZnO NPs, T-Ampicillin, C-Zinc nitrate precursor solution).

The antibacterial activity of ZnO NPs that had been aided by mango seed extract against *Bacillus subtilis* and *E. coli* was studied (Figure 7). Previous research indicated that ZnO NPs demonstrated strong antibacterial action against a wide variety of microorganisms [14,45,46,47]. In agreement, the present study results also exhibited a significant inhibitory effect against the tested pathogens, compared with standard antibiotics. Earlier studies have also shown the ZnO NPs bactericidal activity, indicating that the nanoparticle can completely kill the bacteria [48]. Nevertheless, our study showed that ZnO NPs prepared from mango seed extract possess bacteriostatic properties. Both the tested organisms exhibited a minimum inhibitory concentration (MIC) at 10 μL. The inhibitory effect is indirectly proportional to the size of the nanoparticle. The smaller size of the nanoparticles the greater will be the inhibitory effect [49].

### 2.8. Antioxidant Activity

**Figure 8:**
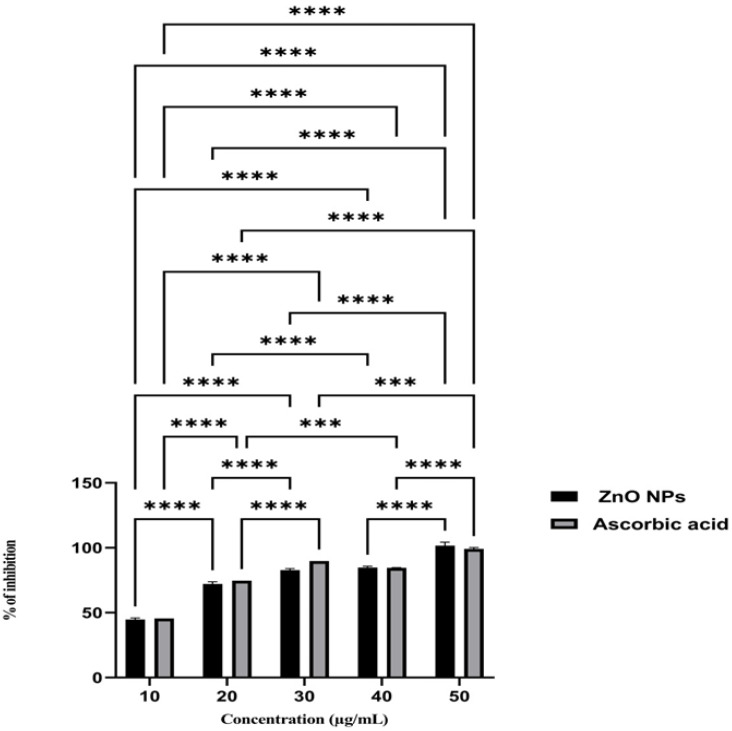
Antioxidant activity of ZnO NPs (ascorbic acid as a standard). Asterisks show a statistically significant difference (****—*p* ˂ 0.0001, ***—*p* ˂ 0.001).

The DPPH experiment assessed ZnO NPs’ antioxidant capabilities. The experimental methodology used 0.1 mM DPPH solution to examine ZnO NPs at 10, 20, 30, 40, and 50 µg (Figure 8). After mixing the solutions together, they were left to sit for 30 min at room temperature and out of the light. After that, absorbance and optical density were both obtained at a wavelength of 517 nm. The reference standard used in this assay was Ascorbic acid. According to the results, the level of DPPH inhibition dramatically increased whenever there was a higher concentration of ZnO NPs. The results that were acquired revealed that the ZnO NPs that were produced had powerful antioxidant capabilities.

## 3. Materials and Methods

### 3.1. Mango Seed Extract Preparation

**Figure 9:**
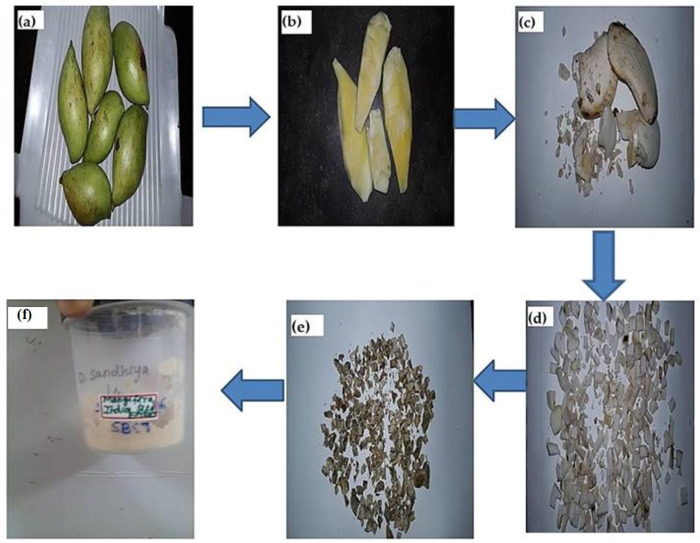
Preparation of mango seed extract. (**a**) Collection of raw mangoes (**b**) Collection of raw mango’s seed part (**c**) Mango seed part was cut into small pieces (**d**) Shade dried (**e**) Collection of shade dried mango’s seed part (**f**) Grounded to obtain coarsely powdered form.

Raw mangoes were purchased and used as a source for harvesting mango seeds. The mango’s endocarp part (seed part) was cut into smaller pieces and shade dried for 5–7 days in Nanobiomedicine Lab, Saveetha Dental College and Hospitals, India. The shade-dried mango seed was ground into coarsely powdered form. A conical flask containing 100 mL of distilled water and 1 g of powdered mango seed. It was continuously stirred using a magnetic stirrer up to 600 rpm. After that, it was placed in the heating mantle at 70 °C and heated for 15–20 min until the hard powder became soft and mushy. Whatman No.1 filter paper was used to filter the fluid. Figure 9 depicts the steps required to make mango seed aqueous extract.

### 3.2. Zinc Oxide Nanoparticle Synthesis

**Figure 10:**
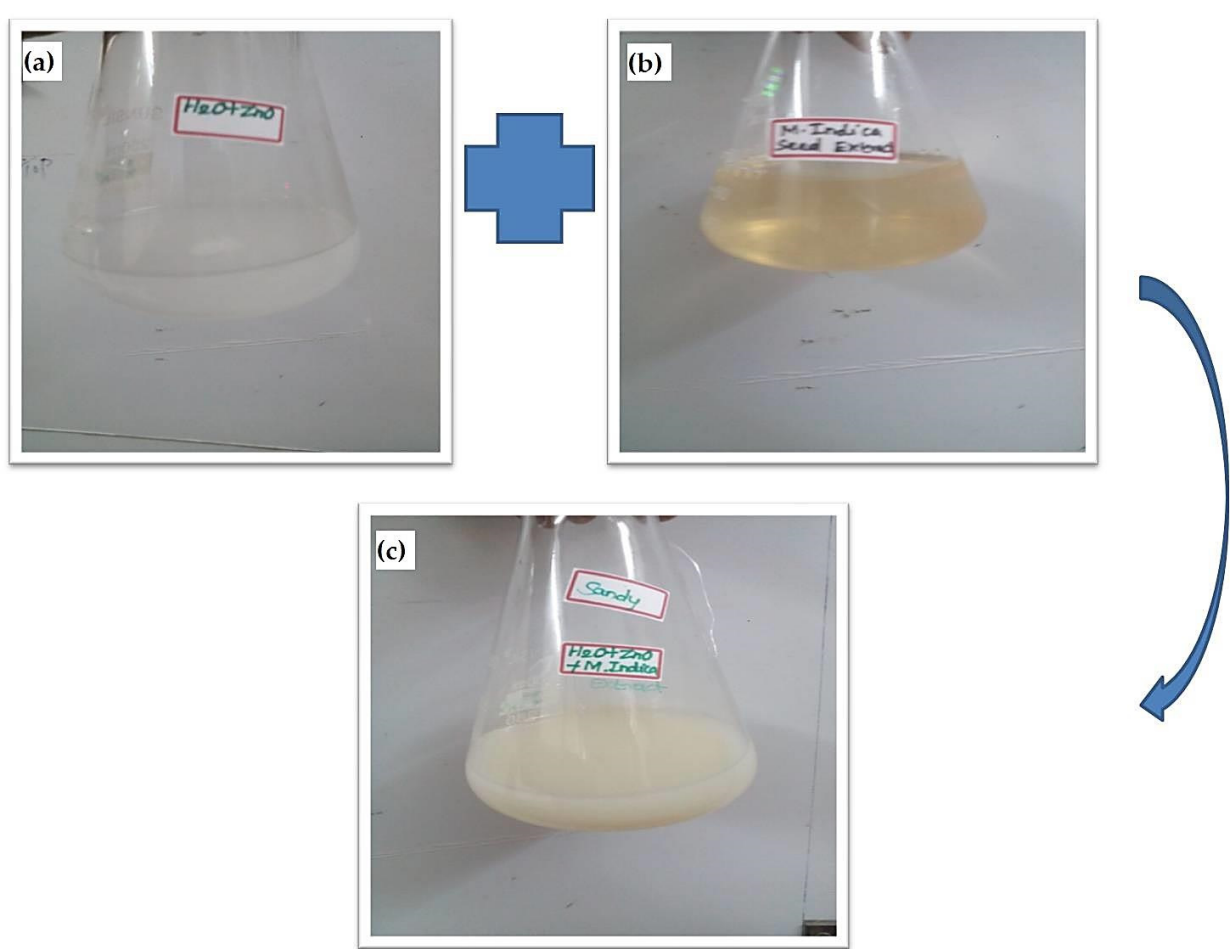
*M. indica* seed extract-based ZnO NP synthesis. (**a**) 10 mM Zinc nitrate precursor solution (**b**) *M. indica* seed extract (**c**) *M. indica* seed extract combined with Zinc nitrate precursor solution.

A 10 mM Zinc nitrate solution was added to 75 mL of distilled water. To that, 25 mL of the ready-to-use seed extract was added, and everything was mixed well. At 30 °C, the solution combination was maintained in an orbital shaker for 15 h. ZnO NPs formed when the solution’s colour changed from white to dark brown after 15 h (Figure 10). The solution was then centrifuged at 8000 rpm for 20 min. After the centrifugation process, the supernatant was discarded, and the pellet was washed twice with deionized water in order to eliminate any remaining residual contaminants. After that, the pellet was extracted from the centrifuge and kept in an oven with heated air at a temperature of 80 °C. Upon drying, the pellet was ground into a powder. This ZnO NPs powder was used for further SEM, EDX, AFM, XRD and FT-IR investigation.

### 3.3. ZnO NPs Characterization

In order to evaluate the UV absorption spectra of the ZnO NPs that were created, a Shimadzu UV spectrophotometer was used to measure the spectrum from 300 to 800 nm. This was done so that the spectra could be used to calculate the UV absorption spectra. An FT-IR spectrum was recorded with a BRUKER alpha 2. In order to determine the nanoparticles’ X-ray diffraction (XRD) pattern, XPERT PRO, PANalytical XRD was used. Using a JEOL-JSM IT 800 model, we performed the SEM-EDAX analysis. Atomic force microscopy (AFM) was used in order to investigate the synthesized ZnO NPs for their size and surface roughness (Nanosuf AGG Switzerland).

### 3.4. Antioxidant Activity

According to the findings of Koleva et al. [50], the 2,2-diphenyl-1-picrylhydrazyl (DPPH) test was used to evaluate the ZnO NPs’ capacity to scavenge free radicals. A DPPH solution of 150 µM in 100 mL of methanol was made. For this experiment, 190 µL of DPPH solution was combined with 10 µL of the synthesized ZnO NPs and varying doses of standard ascorbic acid (10–50 µg/mL). The liquid was let to rest at room temperature and in the dark for 30 min. Instead of a sample or standard, 200 µL of methanol was used in the control blank. The maximum absorbance was found to be at 517 nm. ZnO NPs’ ability to scavenge DPPH radicals was calculated using the following formula.

% free radical scavenging effect = [(Control absorbance − Test absorbance)/Control absorbance] × 100

### 3.5. Antibacterial Activity

Gram-positive *B. subtilis* and Gram-negative *E. coli* strains were used in an experiment to test the antibacterial activity of synthesized ZnO NPs. The experiment was conducted using the agar well diffusion method [41]. The nutritional broth was contaminated with the clinical pathogens and cultivated for a whole day at 37 °C. Cotton swabs made from sterile material were used to apply a suspension of the organisms to be tested on Mueller-Hinton agar (MHA) plates that had been prepared using aseptic methods. With a clean borer, four holes were drilled in each MHA plate. Then, 50 μL of precursor, 50 μL of ZnO NPs and 15 μL of ampicillin were introduced into the bored wells. Afterward, plates were incubated at 37 °C for 12 h. A distinct zone of inhibition surrounded each well after incubation, which could be measured with the ruler.

### 3.6. Statistical Analysis

The value of antioxidant activity was expressed in terms of Mean ± SE for three independent experiments. One-Way ANOVA followed by Tukey post hoc multiple comparison tests were performed to compare and evaluate the data with *p* ≤ 0.05 considered to be significant.

## 4. Discussion

Green nanotechnology employs medicinal plants for the synthesis of metal as well as other nanomaterials that have the potential to be used in the identification and treatment of various diseases/disorders. Metal nanoparticles synthesized from medicinal plants, microbes and other food sources have been shown to be safe and economical. However, environmental sustainability is a concern due to the load on global food security and the scarcity of natural resources [51]. In this context, researchers have initiated to utilize the biowaste materials from various plant and fruit sources to synthesize metal-based and metallic oxide nanoparticles. Several ways for generating ZnO NPs utilizing various plant extracts have been established by researchers [32,33,34,35,36,37,38,39,40,41,42]. We report here on the large - scale production of ZnO NPs by using an aqueous extract of mango seed powder as the raw material. The ZnO NPs were produced by combining a zinc nitrate solution combination with an aqueous extract of mango seeds as the phytoconstituents throughout the synthesis process. The solution’s colour dramatically altered after being incubated for a certain amount of time. The shift in hue is attributed to the production of ZnO NPs and the resulting surface plasmon resonance from the collective excitation of free electrons in the NPs. The qualitative phytoconstituent analysis of aqueous mango seed extract showed the presence of polyphenols, tannins, flavonoids and terpenoids, which might be attributed as responsible for biological properties. These phytochemicals found in the mango seed aqueous extract may also be liable for reducing the Zinc ions to zinc oxide and, thus, nanoparticle preparation.

In this work, we used green synthesis, an environmentally friendly approach to create ZnO NPs from fruit biowaste. A zinc nitrate solution was combined with an aqueous extract of mango seeds in order to produce ZnO NPs. This method was determined to be cheap, quick and safe for the environment. In a previous investigation, ZnO NPs were synthesized using the seeds of the longan fruit. The seed was found to be enriched in catechin and flavonoids [52]. The current investigation also employed Mango seed extract to produce ZnO NPs. The findings demonstrated the emergence of ZnO NPs. ZnO NPs formation was originally seen as a colour shift in the metal solution upon addition of mango seed extract; this observation was subsequently confirmed using further physicochemical techniques. Absorption spectra at 480 nm, typical of ZnO NPs, were observed through UV-Vis spectroscopy. Previous research also reported the UV-Visible absorption peak up to 381 nm using Pomegranate extract [53].

The FT-IR analysis demonstrates the presence of carboxylic, ketone, nitro, ester and ether groups. The XRD analysis predicts seven prominent peaks. The SEM data indicate the presence of spherical and cylindrical nanoparticles between the size range of 40–70 nm and a few rectangular and triangular particles. Recently, a study by Rini et al. (2021) reported that ZnO NPs synthesized using *Ananas comosus* peel extract had a spherical shape [54]. The findings of EDAX indicate the existence of carbon, oxygen and zinc. The morphology of the nanoparticle’s surface is studied using an AFM. In addition, data on the size and surface roughness of the produced nanoparticles are provided. The TEM analysis identified the nanoparticle’s shape as spherical and the size ranging from 40–60 nm. It was reported in a prior research that the average size of the ZnO NPs that were synthesized using *Azadirachta indica* extract was 9.6–25.5 nm, and that their form was spherical [55]. The AFM may be used to take 3D images without causing any damage. The AFM data indicated that the nanoparticles were present in the spherical form [56]. Furthermore, the mango seed extract-assisted ZnO NPs have been demonstrated to possess potent antibacterial and antioxidant properties, as observed from agar well diffusion assay and DPPH free radical scavenging assay.

Even while research has shown that ZnO NPs has a powerful antioxidant capacity, the method by which this occurs is still a mystery. During the green synthesis of nanoparticles, the functional groups of the phytoconstituents have been shown to form a linkage with the ZnO. This linkage may improve the potential of ZnO NPs in free radical scavenging effect [57]. The high redox potential of the ZnO breaks the water molecules into hydroxyl and hydrogen radicals, stabilizing the DPPH free radicals and inhibiting the DPPH effect [58]. According to the findings, there was a considerable increase in the inhibitory capacity of ZnO NPs against the DPPH free radicals that was dose-dependent. It may be deduced from this that the ZnO NPs that were manufactured using the mango seed extract exhibited a substantial amount of antioxidant activity. Furthermore, as was previously indicated, the phytoconstituents’ functional groups adsorbing onto the nanoparticles’ surfaces might be a contributing cause to their inhibitory action on free radicals [59].

One of the most significant issues in the medical field is the incorrect and excessive use of antibiotics, which results in antimicrobial resistance. The persistent emergence of bacterial resistance has raised the need for novel antibiotics. Metal nanoparticles, which have been reported to have potent antibacterial action in a majority of investigations, are considered among the promising as well as novel antibiotic agents [27,28]. Production of new biomedical implants involves the use of metallic nanoparticles in order to prevent any bacterial infections [59]. As a result, one of our goals was to determine whether or not the ZnO NPs that we had manufactured using mango seed extract have any antibacterial properties. In the present experiment, it was discovered that the ZnO NPs that were artificially manufactured have a substantial antibacterial activity. ZnO NPs have been shown to inhibit bacterial growth via a variety of distinct methods, which has led to its use in antibacterial applications. According to a number of studies, metal nanoparticles have the ability to physically interact with the cell wall of bacteria, as well as with sub-cellular components [60]. On exposure to metal nanoparticles, the bacteria’s cell wall undergoes membrane damage due to the adsorption of metal oxide on the cell wall.

The negatively charged surface of the bacteria stimulates electrostatic interactions between strong positive charges such as ZnO (>9) with high isoelectric points [58]. This results in the membrane depolarization effect, which alters the cell wall’s permeability, allowing an easier penetration of the nanoparticles and producing reactive oxygen species (ROS) inside the bacterial cell. The increased ROS production eventually causes oxidative stress and elevates lipid peroxides, thereby degradation of macromolecules and resulting in cell death [61,62]. Ion leaching is another proposed mechanism for the antibacterial effect of metal nanoparticles. The pH and rate of dissolution of ZnO NPs have also been shown to cause inhibition in bacterial cell growth [28,63]. Further, it was also reported that spherical-shaped nanoparticles could easily penetrate the cell wall of the bacteria, thus resulting in cellular membrane damage [48].

## 5. Conclusions

In conclusion, our findings have provided new insights into the biomedical applications of biowastes, such as seeds or peels of medicinal plants, in the preparation of metal nanoparticles. This work showed that ZnO NPs synthesized with the aqueous mango seed were efficient against tested clinical pathogens and exhibited considerable antioxidant activity. The seed-mediated nanoparticles show very good antimicrobial potential against Gram-negative and Gram-positive bacterial isolates, viz., *B. subtilis* and *E. coli*. Therefore, it is possible to propose that this technique of synthesizing nanoparticles by employing plant extracts may assist in discovering new unique active pharmaceutical components and heal the sickness, considering the high nutritional content and cost-effectiveness. However, before clinical investigations can be carried out, it is necessary to conduct in vitro toxicity tests using human cells as well as in vitro and vivo models.

## Figures and Tables

**Table 1:** Elements present in the nanoparticles.

Element	Weight%	Atomic%
C K	64.58	74.14
O K	28.25	24.35
Zn K	7.16	1.51
Totals	100.00	

## Data Availability

Not applicable.

## Sample Availability

Samples of the compounds are available from the authors.
